# Anatomical Studies Evaluating Pediatric Regional Anesthesia: A Scoping Review

**DOI:** 10.3390/children11060733

**Published:** 2024-06-15

**Authors:** Lucas Ferreira Gomes Pereira, Ricardo Vieira Carlos, Albert van Schoor, Adrian Bosenberg, Natália Mariana Silva Luna, Rebeca da Costa Silva, Bianca de Fátima Bertanha, Maria José Carvalho Carmona, Vinícius Caldeira Quintão

**Affiliations:** 1 Discipline of Anesthesiology, Hospital das Clínicas, Faculdade de Medicina, Universidade de São Paulo, São Paulo 05403-010, Brazil; lucas.pereira@fm.usp.br (L.F.G.P.); ricardo.vieira@hc.fm.usp.br (R.V.C.); nmsluna@uni9.edu.br (N.M.S.L.); rebeca.costa0920@uni9.edu.br (R.d.C.S.); bianca.bertanha@fm.usp.br (B.d.F.B.); maria.carmona@fm.usp.br (M.J.C.C.); 2 Instituto da Criança e do Adolescente, Hospital das Clínicas HCFMUSP, Faculdade de Medicina, Universidade de São Paulo, São Paulo 05403-000, Brazil; 3 Department of Anatomy, School of Medicine, Faculty of Health Sciences, University of Pretoria, Pretoria 0085, South Africa; albert.vanschoor@up.ac.za; 4 Department of Anesthesia and Pain Management, Seattle Children’s Hospital, University of Washington, Seattle, WA 98195, USA; adrian.bosenberg@seattlechildrens.org; 5 Department of Medicine, Universidade Nove de Julho (UNINOVE), São Paulo 03155-000, Brazil

**Keywords:** nerve block, regional, cadaver, regional anesthesia, pediatrics, pediatric anesthesia

## Abstract

Background: Pediatric regional anesthesia has been driven by the gradual rise in the adoption of opioid-sparing strategies and the growing concern over the possible adverse effects of general anesthetics on neurodevelopment. Nonetheless, performing regional anesthesia studies in a pediatric population is challenging and accounts for the scarce evidence. This study aimed to review the scientific foundation of studies in cadavers to assess regional anesthesia techniques in children. Methods: We searched the following databases MEDLINE, EMBASE, and Web of Science. We included anatomical cadaver studies assessing peripheral nerve blocks in children. The core data collected from studies were included in tables and comprised block type, block evaluation, results, and conclusion. Results: The search identified 2409 studies, of which, 16 were anatomical studies on the pediatric population. The techniques evaluated were the erector spinae plane block, ilioinguinal/iliohypogastric nerve block, sciatic nerve block, maxillary nerve block, paravertebral block, femoral nerve block, radial nerve block, greater occipital nerve block, infraclavicular brachial plexus block, and infraorbital nerve block. Conclusion: Regional anesthesia techniques are commonly performed in children, but the lack of anatomical studies may result in reservations regarding the dispersion and absorption of local anesthetics. Further anatomical research on pediatric regional anesthesia may guide the practice.

## 1. Introduction

Regional anesthesia in pediatric anesthesia has gradually grown due to the widespread use of multimodal and opioid-sparing analgesia [1,2]. The recent concern over the potentially harmful effects of general anesthetics on neurodevelopment has further contributed to pediatric regional anesthesia development [3].

Although the Bier technique has been described in children since 1899 [4], and peripheral nerve blocks have been reported since 1963 in the pediatric population by Taylor et al. [5], there are few clinical pediatric studies due to the inherent challenges associated with performing clinical studies in this population [6,7].

The Pediatric Regional Anesthesia Network has been investigating the practice, risks, and incidence of complications since 2007. According to the data presented, pediatric regional anesthesia is a safe practice with a relatively low risk of complications [8,9,10]. In contrast to the many anatomical studies developed in adults with a focus on regional anesthesia, studies on pediatric cadavers are still scarce.

This scoping review aims to create a descriptive summary of the included studies and identify gaps in the literature on anatomical studies in pediatric cadavers used to evaluate regional anesthesia techniques.

This review was based on the following research questions: Are there anatomical studies of peripheral nerve blocks in the pediatric population? Based on the studies published in cadavers, what is the existing evidence of anatomic studies for pediatric regional anesthesia techniques?

## 2. Methods

This scoping review strictly adhered to the Preferred Reporting Items for Systematic Reviews and Meta-Analyses extension for scoping review (PRISMA-ScR, Table S1) [11]. The review protocol was registered on the Open Science Framework: https://osf.io/p3u6h/ accessed on 4 April 2024.

The systematic search strategy was performed on the following databases: MEDLINE (PubMed), EMBASE, and Web of Science. Additionally, we manually searched the list of references of the articles selected. We used multiple combinations of the following MeSH terms: “Anatomy, Regional”, “Cadaver”, and “Nerve Block”. The complete search strategies are provided in the Table S2.

Following the PICOs strategy, our study’s target population was pediatric cadavers (under 18 years of age), who had undergone peripheral regional blocks which were analyzed during anatomical studies to assess the dispersion of the injected solutions and/or the anatomy of each particular block. There was no comparison group.

### 2.1. Selection of Studies

The studies considered eligible for inclusion in this scoping review were human anatomical studies evaluating any type of peripheral block in the pediatric population, including zero to 18 years of age. Central neuraxial block studies were not included, as well as studies in other species and articles without full text available, with no publication date, or in a language different from English. The reference lists of articles included in the review were screened for additional papers.

All identified records were uploaded to EndNote v.20 software (Clarivate Analytics, Philadelphia, PA, USA), and duplicates were removed. Titles and abstracts were screened by two independent reviewers (L.F.G.P. and V.C.Q.) to assess whether they met the inclusion criteria for the review.

Relevant papers were retrieved, and their citation details were uploaded into Rayyan (Qatar Computing Research Institute, Doha, Qatar). Full-text studies that did not meet the inclusion criteria were excluded, and any disagreements between reviewers were resolved through discussion or with a third reviewer (R.V.C.).

### 2.2. Data Extraction

Two independent reviewers (L.F.G.P. and V.C.Q.) used a data extraction tool developed by the reviewers (Table S3) to extract the summarized data from papers included in the scoping review. The data extracted included specific details about the year of the publication, the population studied, the block studied, cadaver characteristics, the block assessment, and the results relevant to the review question.

Any reviewer disagreements were resolved through discussion with a third reviewer (R.V.C.). Where required, authors of papers were contacted by the contact email to request missing data.

### 2.3. Data Analysis and Presentation

The data collected from the selected studies were divided into two tables containing the primary data with the most significant emphasis on the block studied and how the block was assessed. Data were extracted from selected studies to an extraction chart, and the following information was included in the tables: publication year, name of authors, country where the study was performed, anesthesia block investigated, population, cadaver characteristics, block evaluation, results, and conclusion.

### 2.4. Secondary Analysis

As observed during the study selection phase, many anatomical studies related to anesthetic blocks were found in other populations and species. This interesting fact yielded a further analysis for this review, which will be presented as an infographic.

## 3. Results

### 3.1. Search

**Figure 1:**
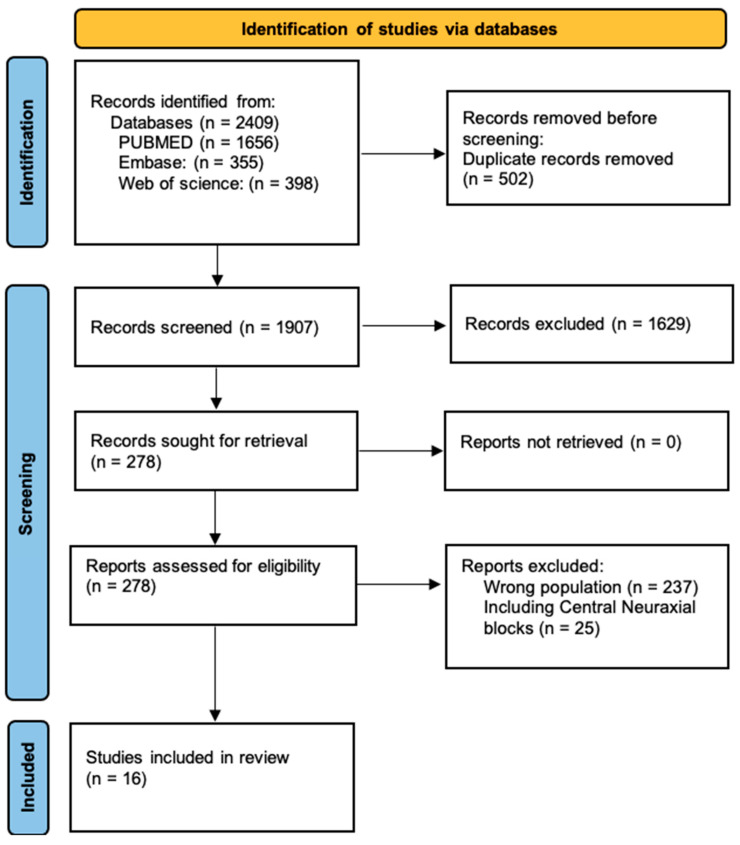
Preferred Reporting Items for Systematic Reviews and Meta-Analyses (PRISMA) flow diagram for the present scoping review.

The systematic database search found 2409 studies. After removing 502 duplicates, 1907 studies were screened based on the title and abstract, and 1629 manuscripts were excluded. The full texts of 278 studies were assessed for eligibility, and 262 were excluded. The reasons for exclusion were 237 studies with the wrong population and 25 studies including central neuraxial blocks in the anatomical studies. A total of 16 studies were included in the review, and the study selection process is illustrated in the PRISMA flowchart diagram (Figure 1).

The studies selected were published between 1995 and 2024. The types of blocks in the studies were erector spinae plane blocks [12,13,14], ilioinguinal/iliohypogastric nerve blocks [15,16], sciatic nerve blocks [17,18], maxillary nerve blocks [19], paravertebral blocks [20], femoral nerve blocks [21], radial nerve blocks [22], greater occipital nerve blocks [23,24], infraclavicular brachial plexus blocks [25], infraorbital nerve blocks [26], and dorsal penile nerve blocks [27].

The included studies were published in nine different journals, with the journal with the highest number of publications being *Pediatric Anesthesia*, which published six studies in total.

**Figure 2:**
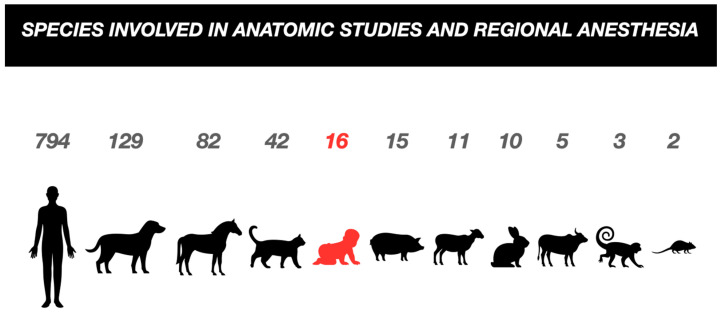
Infographic comparing the studied species, with the pediatric population highlighted in red.

As part of a secondary analysis during the selection of studies, we compared regional anesthesia anatomical studies in different populations. As a result, adult humans comprised the studied population with the highest number of studies, 794 articles, followed by studies in dogs, horses, and cats, and only then in the pediatric human population. The data found in the search are summarized in the infographic below (Figure 2).

Our comparison-based selection resulted in few studies in the pediatric population, probably due to the challenges inherent to performing research involving this population.

### 3.2. Study Characteristics

Table 1 and Table 2 depict and summarize the data collected in the studies. Table 1 describes the studies according to the population studied in the publications included in the scoping review.

Table 2 summarizes the assessment performed for each anesthesia block, results, and conclusions from the studies included.

## 4. Discussion

This scoping review analyzed regional anesthesia using cadavers in the pediatric population. Acknowledging how the topic has already been studied, which conclusions were consistent, and the existing knowledge gaps in the pediatric population are essential to the progress of pediatric regional anesthesia research.

When it comes to pediatric anesthesia, a committee with the council and board of the pediatric anesthesia societies of the United Kingdom, Ireland, New Zealand, and Australia chose pediatric regional anesthesia as the second main topic of interest for new research. And new discoveries for this population should not be based on the concept of children being “little adults” [28,29]. The differences in regional anesthesia between these populations are profound, from the anatomy that varies according to the age of the patient and the thinner muscles, fascia, and connective tissues compared to adults, to questions of pharmacodynamics and pharmacokinetics [29].

The subject was primarily studied through the performance of anatomical measurements (12 studies out of the 16 included) rather than the actual simulated performance of blocks in cadavers. Studies with block simulations investigated interfascial block techniques (erector spinae plane and paravertebral blocks), and in most of the studies, the dispersion was recorded and correlated with the injected volume [12,13,14,20].

In studies that aimed to simulate the block’s performance, injected solution dispersion was assessed through imaging such as a CT scan [14] or even ultrasonography [13]. Along with imaging tests, solution dispersion was confirmed by dissection after performing blocks.

The peripheral nerve blocks more frequently assessed by anatomical studies in the pediatric population were erector spinae plane block, with three studies included [12,13,14], and ilioinguinal/iliohypogastric nerve block [15,16] and greater occipital nerve block [23,24], with two studies included each.

When we analyzed the age of the subjects in the manuscripts in this scoping review, we found that most cadavers were below the first year of life. This age group has anatomical particularities, and there have been few clinical studies on this population due to the developmental stages that are still to come. This may justify the greater interest in research rather than just extrapolating the technique used in adults.

Developing research in vulnerable populations, such as pediatrics, is a challenging task. This can be seen in the fact that 70–90% of the drugs prescribed for this population are used off-label, showing that the lack of data derived from research in children is not limited to the subject of pediatric regional anesthesia but extends to all areas of medicine [30].

Taking into account the years of the publications included, we found a range between 1995 and 2024. We must bear in mind the technological availability of the time, mainly in the evolution of ultrasound and the study of sonoanatomy, both for performing anesthetic blocks and for evaluating the dispersion of the blocks performed.

Considering the conclusions of the selected studies, we realized that they agreed that, due to anatomical and physiological differences between adults and children, it would be inappropriate to extrapolate the findings obtained from an adult sample to the pediatric population.

This study can serve as a basis and guide for new anatomical studies on regional blocks in the pediatric population. In addition, it summarizes the results found in each anatomical study, compressing data on pertinent anatomical details for each block and on the evaluation of block dispersion, especially in studies in which there was simulation during the anatomical study.

After examining the literature, we observed gaps, such as the need to perform studies focusing on peripheral nerve block techniques commonly employed in pediatric patients, such as rectus sheath block and transversus abdominis plane block. There is a need to simulate blocks in anatomical studies to assess nerve block techniques, more accurate imaging tests to assess injected solution dispersion, and the possibility of analysis using Magnetic Resonance Imaging.

The main limitation of our review is the age of the existing anatomical studies, most of which are of the neonatal population. In addition, we could not ensure the state of preservation of the cadavers studied, including premature cadavers, using blocks guided by anatomical landmarks and the heterogeneity of the studies included.

### Issues Related to Cadaver Preservation/Preparation

Different cadaver preparation and preservation types can influence the results in anatomical studies. In addition, characteristics of in vivo patients absent from cadavers also influence the evaluation of blocks, especially regarding their dispersion [23].

Such characteristics, which do not allow the cadaver model to mimic the clinical scenario fully, include blood circulation and muscle contraction (important points for diffusion), respiratory and body movement, postmortem turgor, and the concern that the area of dispersion will increase during dissection [31,32].

The temperature at which the cadaver is preserved also interferes with connective tissue viscosity. Hyaluronic acid (a key viscosity component) is susceptible to temperature change and acidosis. Dropping to a pH of 6.6 and decreasing cadaver temperature by 2 °C increases viscosity by up to 20% [31,33].

In addition to hyaluronic acid, the water in the interfascial connective tissue is considerably reduced, leading to high viscosity and resistance to injection in the fascial plane. Such biochemical and biomechanical characteristics may influence the extent and dispersion pattern of the injected solution, especially in interfascial blocks, depending on the type of preservation used [34].

## 5. Conclusions

Peripheral nerve blocks in pediatric patients are extensively used components of the opioid-sparing strategy. Anatomical studies on the subject are scarce, with a high diversity of studies that can still be developed. There are peripheral blocks that have not yet been addressed by studies on pediatric cadavers, thus providing a good opportunity for research in pediatric anesthesiology.

Most regional anesthesia techniques are based on the extrapolation of data from blocks performed in adults, which presents uncertainties to the practitioner regarding the dispersion and absorption of local anesthetics, toxic doses, and points of needle insertion. Thus, further anatomical studies are required in the pediatric population.

## Figures and Tables

**Table 1:** Study characteristics according to population.

Year	Authors	Country	Block Studied	Population	Cadaver Characteristics
2020	Govender et al. [12]	South Africa	ESP Block	2 neonates	Fresh unembalmed preterm neonatal cadavers subject to cryopreservation; cadaver 1: 1.6 kg, cadaver 2: 0.6 kg.
2020	Govender et al. [13]	South Africa	ESP Block	9 neonates	Fresh unembalmed preterm neonatal cadavers subject to cryopreservation. Weight: 0.7 kg, 1.2 kg, 1.35 kg, 1.7 kg, 1.8 kg, 2 kg, 2.6 kg, 2.95 kg, 3.4 kg.
2020	Govender et al. [14]	South Africa	ESP Block	1 neonate	Fresh unembalmed preterm neonatal cadaver subject to cryopreservation. Weight: 2.3 kg.
2005	van Schoor et al. [15]	South Africa	Ilioinguinal iliohypogastric block	25 infants and neonates	Embalmed cadavers (mean weight = 2.2 kg; mean height = 45.6 cm).
2013	Van Schoor et al. [16]	South Africa	Ilioinguinal iliohypogastric block	54 neonates	Embalmed cadavers (mean length: 43 cm; mean weight: 1.9 kg).
2014	Reinoso-Barbero et al. [17]	Spain	Sciatic nerve block	13 adults and 11 newborns	Embalmed cadavers in a solution of phenol and glycerin (mean age = 6 days; mean weight = 3.3 kg; mean height = 48.4 cm).
2017	Acar et al. [18]	South Africa	Sciatic nerve block	20 neonates	Formalin-fixed preterm and full-term cadavers (mean weight = 1.2 kg; mean height = 40 cm).
2014	Prigge et al. [19]	South Africa	Maxillary nerve block	30 pediatrics	10 dried pediatric skulls and 30 formalin-fixed pediatric cadavers. Group 1—Neonate cadavers; Group 2—28 days to 1 year.
2014	Albokrinov et al. [20]	Ukraine	Paravertebral block	20 infants	Fresh, unembalmed infant cadavers (median age = 7 months; median body mass 4550 g).
2022	Cihan et al. [21]	Turkey	Femoral nerve block	30 newborns	Formalin-fixed cadavers, ranging in age from 9 to 40 weeks.
2012	Gupta et al. [22]	India	Radial nerve block	30 newborns	Spontaneously aborted fetuses with age between 32 and 40 weeks.
2019	Prigge et al. [23]	South Africa	Greater occipital nerve block	6 pediatrics	Pediatric formalin-fixed cadavers (mean age 51.4 days and one cadaver of 2 years).
2023	Yagmurkaya et al. [24]	Turkey	Greater occipital nerve and the third occipitalnerve extend block	15 fetal cadavers (3rd trimester)	The immersion method used 10% formalin, and the gestational ages were determined depending on the crown–rump length.
2009	Bosenberg et al. [25]	South Africa	Infraclavicular brachial plexus block	52 neonates and 81 adults	Mean weight −1.94 kg ± 1.62; mean height—0.43 m ± 0.08.
1995	Bosenberg et al. [26]	South Africa	Infraorbital nerve block	15 neonates	Fresh stillborn cadavers (mean weight = 2.85 kg.
2024	Zadrazil et al. [27]	Austria	Dorsal penile nerve block	3 pediatric cadaveric specimens	Fixed with Thiel’s method.

ESP, erector spinae plane block.

**Table 2:** Assessment of the anesthesia block, results, and conclusions for each study included in the review.

Year	Authors	Block Studied	Assessment of the Block	Results	Conclusion
2020	Govender et al. [12]	ESP block	The block was replicated using methylene blue dye at vertebral levels T5 using 0.5 mL in cadaver 1 and in T8 using 0.2 mL in cadaver 2. The spread of dye was tracked on the ultrasound and confirmed on dissection.	Cadaver 1—spread from vertebral levels T3 to T6. Cadaver 2—spread from vertebral levels T7 to T11. Methylene blue was found in the paravertebral and epidural spaces, staining both dorsal and ventral rami of spinal nerves T2 to T12.	The dye injected spread to the paravertebral, epidural, and intercostal spaces and over multiple dermatomal levels from T2 to T12. More studies are needed to compare ESP over paravertebral and epidural spaces in this population.
2020	Govender et al. [13]	ESP block	The block was performed ultrasound-guided bilaterally using 0.1 mL/kg of iodinated contrast dye at vertebral levels T5 and T8. Dissections were performed 30 min after injection.	14 blocks were successful, and 4 blocks were incomplete or failed. The contrast was found in paravertebral, intercostal, and epidural spaces, including over the neural foramina. The needle direction or entry side did not influence the spread.	The contrast injected was found in the paravertebral, intercostal, and epidural spaces over an average of 5 dermatomal levels using 0.1 mL/kg of solution.
2020	Govender et al. [14]	ESP block	The ultrasound-guided block was performed bilaterally using 2.3 mL (1 mL/kg) of iodinated contrast dye. It was carried out at vertebral levels T8 on the right-hand side and T10 on the left-hand side, followed by a CT scan after 20 min.	The contrast was observed to spread over three dermatomal levels from T6 to T9 on the right side and over four dermatomal levels from T9 to T12 on the left side. Additionally, it spread over the costotransverse ligament into the paravertebral space, with no spread detected in the epidural space.	The contrast dye was found in the paravertebral space at four dermatomal levels, suggesting an approximate volume of 0.5–0.6 mL per dermatome.
2005	van Schoor et al. [15]	Ilioinguinal iliohypogastric block	The aim of the study was to use dissection to determine the precise anatomical position of the nerves relative to the ASIS.	The distance from left ilioinguinal nerve—1.9 ± 0.9 mm; Right ilioinguinal nerve—2.0 ± 0.7 mm. Left iliohypogastric nerves to the ASIS—3.3 ± 0.8 mm; right iliohypogastric nerves—3.9 ± 1.0 mm.	It is suggested that the high failure rate of the nerve block in this population could be attributed to a lack of knowledge of the anatomy of these nerves. The insertion point is approximately 2.5 mm from the ASIS, on a line connecting the ASIS to the umbilicus.
2012	van Schoor et al. [16]	Ilioinguinal iliohypogastric block	The position of the ilioinguinal and iliohypogastric nerves in relation to the ASIS was studied by dissecting neonate cadavers.	- Distance from the ilioinguinal nerve to ASIS: 2.2 ± 1.2 mm. - Distance from the iliohypogastric nerve to ASIS: 3.8 ± 1.3 mm. The recommended needle insertion site is 3.0 mm from ASIS. There is a strong correlation between the needle insertion point and the neonate’s weight.	A linear regression formula was determined to calculate the needle insertion distance (mm) based on the weight (kg): distance (mm) = 0.6 × weight (kg) + 1.8. This formula serves as a guideline for the position of the ilioinguinal and iliohypogastric nerves.
2014	Reinoso-Barbero et al. [17]	Sciatic nerve block	The SN, TN, and CPN were dissected, and the distances were measured from the origin of the SN to its division and from there to the popliteal crease. The aim was to clarify the anatomical variability.	The distance from the popliteal crease to the sciatic nerve division was shorter in neonates than in adults (1.04 ± 0.9 cm vs. 5.6 ± 5.1 cm). The sciatic nerve divided proportionally farther down the leg in neonates compared to adults (86 ± 13 vs. 74 ± 15).	The site of the SN division shows high variation from birth, being much closer to the popliteal fossa in newborns than in adults. Its position still varies, so ultrasound guidance for this block is highly recommended.
2017	Acar et al. [18]	SN block	Neonatal cadavers were dissected to locate the sciatic nerve (SN) and measure the distances from the nerve to the greater trochanter of the femur and the tip of the coccyx.	The SN was located 14.9 ± 2.4 mm to the side of the coccyx tip, and the distance from the greater trochanter to the coccyx tip was 27.3 ± 4.0 mm. There was a weak correlation between these distances and the weight and height of the population.	In neonates, the sciatic nerve is typically located midway between the greater trochanter of the femur and the tip of the coccyx. These findings may assist in guiding ultrasound visualization.
2014	Prigge et al. [19]	Maxillary nerve block	To determine the best approach for blocking the maxillary nerve within the pterygopalatine fossa, this study evaluated the needle course to block the nerve as it exits the skull at the foramen rotundum and compared it with two techniques.	The suprazygomatic approach to the pterygopalatine fossa did not show any statistical significance. However, there were statistically significant differences between the suprazygomatic approach, which enters from the midpoint on the lateral border of the orbit, and the infrazygomatic approach, which enters at a point on a vertical line along the lateral orbit wall.	The suprazygomatic approach from the frontozygomatic angle yields the most consistent results in pediatric cadavers. The needle can be advanced horizontally for approximately 20 mm in neonates and 30 mm for infants younger than one year.
2014	Albokrinov et al. [20]	PVB	To assess the dispersion of dye in the paravertebral space of infants, evaluate the effectiveness of a single-injection paravertebral block, and determine the ideal volume needed to cover the paravertebral segments by injecting methylene blue dye at the T12 vertebral level and dissecting the cadaver.	The volume and number of segments are strongly correlated. In all cadavers, the T11, T12, and L1 nerve roots were stained. An optimal volume of 0.2 to 0.3 mL per kg was found to involve the T10-L1 segments. We observed an anterior and contralateral spread of dye, and the incidence correlated with the volume of dye.	A single thoracolumbar paravertebral injection can be administered for lower abdominal anesthesia using 0.2 to 0.3 mL/kg of local anesthetic for a single injection.
2022	Cihan et al. [21]	FN block	To determine an adequate area for safe blocking. The exit point of the femoral nerve block and two measurements were taken: the level of the FN division (high-level division—proximal of the IL; mid-level division—on the level of the IL; and lower-level division—distal of the IL) and its relationship with surrounding structures.	In the study, a strong correlation was found between fetal development and limb division. The researchers observed high-level limb division in 6 fetal cadavers, mid-level division in 33 cadavers, and lower-level division in 21 cadavers. Additionally, two formulas were developed based on the findings. Formula 1 calculates the distance between the FN and the ASIS as −1.221 + 0.408 × gestational age (weeks), while Formula 2 computes the distance between the FN and the PT as −1.321 + 0.621 × gestational age (weeks).	Gestational age-based regression equations from the study indicated that the block site could be effectively identified using palpable bone structures to increase the success of the blockade.
2012	Gupta et al. [22]	Radial nerve block	To explore the terminal branches of the SBRN in fetuses, 30 cadavers were dissected, and measurements were made of the relationship of cephalic veins and their tributaries, as well as the branching patterns found.	Three distinct patterns of innervation were observed: Type 1 (66.7%): SBRN innervated the lateral two-and-a-half digits. Type 2 (23.3%): SBRN innervated the lateral three digits. Type 3 (10%): SBRN innervated the lateral three-and-a-half digits. Additionally, the cephalic vein intersected more than twice along its course.	This detailed branching pattern will improve the success rate of radial nerve blocks and minimize postoperative complications associated with injuries.
2019	Prigge et al. [23]	Greater occipital nerve block	Developing a technique to block the greater occipital nerve in children by evaluating the anatomy of the nerve and the occipital artery. Obtaining measurements between the nerve and selected landmarks.	The occipital nerve was located, on average, 22.6 ± 5.6 mm from the external occipital protuberance. The average distance of the medial three fingers measured at the proximal interphalangeal joint was 20.4 ± 4.0 mm, and there was a strong correlation between these measurements. In 83.3% of cases, the occipital artery was positioned laterally to the nerve at the trapezius muscle hiatus.	The greater occipital nerve can be blocked around 23 mm from the external occipital protuberance, medially to the occipital artery. This distance is equivalent to the width of the three middle fingers at the proximal interphalangeal joint.
2023	Yagmurkaya et al. [24]	Greater occipital nerve and the third occipital nerve extend block	Palpation identified bone landmarks for reference, and measurements were taken prior to dissection. The location, relationship, and variation of the nerves and muscles were observed.	The greater occipital nerve (GON) was observed to pass through the aponeurosis of the trapezius on the superficial plane in all fetal cadavers. In the deep plane, it was noted that the nerve only extended from the medial 1/3 of the semispinalis capitis on the right side without piercing the muscle in 3.3% of cases, while it pierced the muscle in 96.7% of cases in the total fetal cadavers studied.	The effective area for nerve block and other invasive approaches in the suboccipital region was approximately 2–2.5 cm below the RL and 0.5–1 cm lateral to the midline.
2009	Bosenberg et al. [25]	Infraclavicular brachial plexus block	To determine the relationship between the brachial plexus in the axilla and the coracoid process (CP) and an improved needle insertion site for the infraclavicular block using the CP and the xiphoid process (XS) as landmarks.	- Mean distance between the CP and the lateral cord of the brachial plexus: 5.26 mm - Mean distance between the CP and the medial cord of the brachial plexus: 10.05 mm - Mean distance between CP and needle insertion: 7.66 mm. The distance between CP and the needle insertion point revealed a strong correlation.	Lack of knowledge about differences in distances from bony landmarks and the relative depth of the brachial plexus can lead to complications or failed blocks.
1995	Bosenberg et al. [26]	Infraorbital nerve block	To determine the location of the infraorbital nerve in neonatal cadavers and identify landmarks for the infraorbital nerve. Measurements were taken from the infraorbital foramen to the base of the alae nasi, palpebral fissure, and the angle of the mouth.	The average distance from the infraorbital nerve to the alae nasi was 7.7 mm (SD 1.3) on the left and 7.5 mm (SD 0.8) on the right. The nerve was located halfway along a line from the angle of the mouth to the midpoint of the palpebral fissure, approximately 15.5 ± 1.5 mm from the angle of the mouth.	The described landmarks are easily identifiable, and we have successfully applied them in clinical situations. Additional studies are necessary to validate this point.
2024	Zadrazil et al. [27]	Dorsal penile nerve block	To examine the anatomy of the dorsal penile nerve block, dissections were performed to analyze the relevant structures.	The anatomical studies on the three dissected cadaveric specimens gave us the confidence and anatomical knowledge to position the needle tip near the deep neurovascular bundle of the penis.	The feasibility of pediatric penile surgical interventions in spontaneously breathing patients is ensured by precise execution of the penile root block and a thorough understanding of all anatomical structures and potential challenges.

ESP, erector spinae plane block; CT, Computed Tomography; ASIS, Anterior Superior Iliac Spine; SN, sciatic nerve; TN, Tibial Nerve; CPN, Common Peroneal Nerve; PVB, paravertebral block; IL, Inguinal Ligament; FN, femoral nerve; PT, Pubic Tubercule; SBRN, Superficial Branch of the Radial Nerve; CP, coracoid process; XS, Xiphisternal Joint; SD, Standard Deviation.

## Data Availability

Not applicable.

## Supplementary Materials

The following supporting information can be downloaded at https://www.mdpi.com/article/10.3390/children11060733/s1: Table S1: PRISMA-ScR checklist; Table S2: Complete search strategies; Table S3: Data extraction tool.
